# Optical Detection of CoV-SARS-2 Viral Proteins to Sub-Picomolar Concentrations

**DOI:** 10.1021/acsomega.1c00008

**Published:** 2021-02-23

**Authors:** Tamsyn Stanborough, Fiona M. Given, Barbara Koch, Campbell R. Sheen, André Buzas Stowers-Hull, Mark R. Waterland, Deborah L. Crittenden

**Affiliations:** † Biomolecular Interaction Centre and School of Physical and Chemical Sciences, University of Canterbury, Christchurch 8140, New Zealand; ‡ Protein Science and Engineering, Callaghan Innovation, Christchurch 8140, New Zealand; ¶ MacDiarmid Institute for Advanced Materials and Nanotechnology, Massey University, Palmerston North 4442, New Zealand

## Abstract

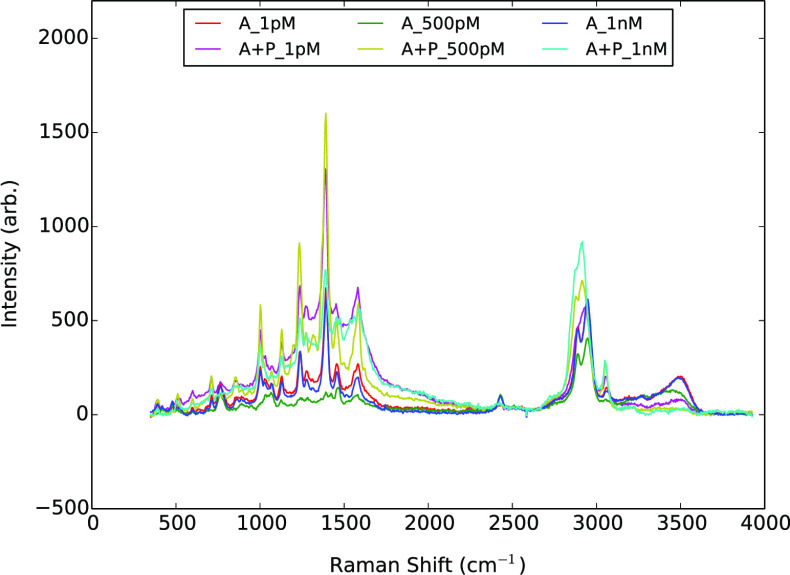

The emergence of a new strain of coronavirus in late 2019, SARS-CoV-2, led to a global pandemic in 2020. This may have been preventable if large scale, rapid diagnosis of active cases had been possible, and this has highlighted the need for more effective and efficient ways of detecting and managing viral infections. In this work, we investigate three different optical techniques for quantifying the binding of recombinant SARS-CoV-2 spike protein to surface-immobilized oligonucleotide aptamers. Biolayer interferometry is a relatively cheap, robust, and rapid method that only requires very small sample volumes. However, its detection limit of 250 nM means that it is not sensitive enough to detect antigen proteins at physiologically relevant levels (sub-pM). Surface plasmon resonance is a more sensitive technique but requires larger sample volumes, takes longer, requires more expensive instrumentation, and only reduces the detection limit to 5 nM. Surface-enhanced Raman spectroscopy is far more sensitive, enabling detection of spike protein to sub-picomolar concentrations. Control experiments performed using scrambled aptamers and using bovine serum albumin as an analyte show that this apta-sensing approach is both sensitive and selective, with no appreciable response observed for any controls. Overall, these proof-of-principle results demonstrate that SERS-based aptasensors hold great promise for development into rapid, point-of-use antigen detection systems, enabling mass testing without any need for reagents or laboratory expertise and equipment.

## Introduction

In late 2019, reports of a novel pneumonia of unknown origin emerged from Wuhan, China.^1^ Since then, the novel coronavirus (SARS-CoV-2) that causes the disease COVID-19 has circulated around the world, leading to a global pandemic.^2^ In November 2020, promising results from clinical trials of COVID-19 vaccines were reported,^3,4^ raising hopes that the “beginning of the end” may be in sight.

In the interim, and in the early stages of any future pandemics, rapid diagnosis and case management is the only practical way to prevent or slow the spread of disease.^5,6^ Laboratory-based real-time quantitative polymerase chain reaction (RT-qPCR) assays are the gold standard testing method, due to their sensitivity (low rates of false negatives) and selectivity (no false positives).^6^ However, RT-qPCR testing has some limitations; it requires substantial time and expertise to collect samples, process them, and return results and can consume substantial quantities of molecular biology reagents, leading to shortages.^7^ It has been widely recognized that optical and/or electronic sensing technologies may hold the key to the development of rapid, high-throughput, easy-to-use, point-of-care diagnostics.^8−11^ However, to date, there have only been a handful of studies that report detection of SARS-CoV-2 viral particles or proteins at biologically relevant concentrations.^12−15^

Of these, two are based upon detecting changes in electrical conductivity upon binding of spike protein antigens to antibodies immobilized on the surface of a graphene-based field-effect transistor^12^ or antibody-functionalized gold nanoparticles deposited on a conductive ITO electrode.^13^ These studies report detection limits of 1 fg/mL (=0.013 fM assuming a molecular weight of 76,500 kDa for the analyte as reported by the supplier) and 1 fM for recombinant spike protein in phosphate-buffered solution, respectively. In biological media—clinical transport medium and spiked saliva samples—detection limits increase 100-fold.

Two optical sensing systems have also been developed; one is based upon surface plasmon resonance (SPR), and another is based upon surface-enhanced Raman spectroscopy (SERS).^14,15^ The SERS device detects changes in the Raman spectra of angiotensin-converting enzyme 2 (ACE2) protein upon antigen binding, but these spectral changes are quite subtle and variable.^14^ When benchmarked against RT-qPCR results, positive and negative wastewater samples fall within substantially overlapping distributions, regardless of how the spectral change is measured. To the best of our understanding, the SPR biosensor is not a conventional SPR assay, i.e. does not rely on detecting plasmonic resonance changes directly. Instead, it appears to detect changes to the optical properties (optical transmissibility) of nanostructured systems that result from changes to the plasmonic resonance structure upon analyte binding.^16^ The authors report a detection limit of 30,000 viral particles per mL (0.05 fM) for SARS-CoV-2 “pseudovirus” binding directly to a nanoplasmonic sensor material functionalized with SARS-CoV-2 antibodies, dropping to 30 viral particles per mL (0.05 aM) if subsequently treated with ACE2-functionalized gold nanoparticles.^15^

In this work, we report three new photonic systems for sensing SARS-CoV-2 antigens and assess their detection limits. All three are based upon functionalizing surfaces with compact, structured DNA aptamers that bind selectively and specifically to the SARS-CoV-2 spike protein receptor binding domain^17^ and detecting changes in surface properties, either refractive index (biolayer interferometry and surface plasmon resonance) or the vibrational spectrum of the probe aptamer (surface-enhanced Raman spectroscopy). We note that the strategies employed here may be useful for improving the performance, longevity, cost, and/or ease of fabrication of other sensing systems reported in the literature.^8−10^

## Results and Discussion

### Biolayer Interferometry (BLI)

**Figure 1:**
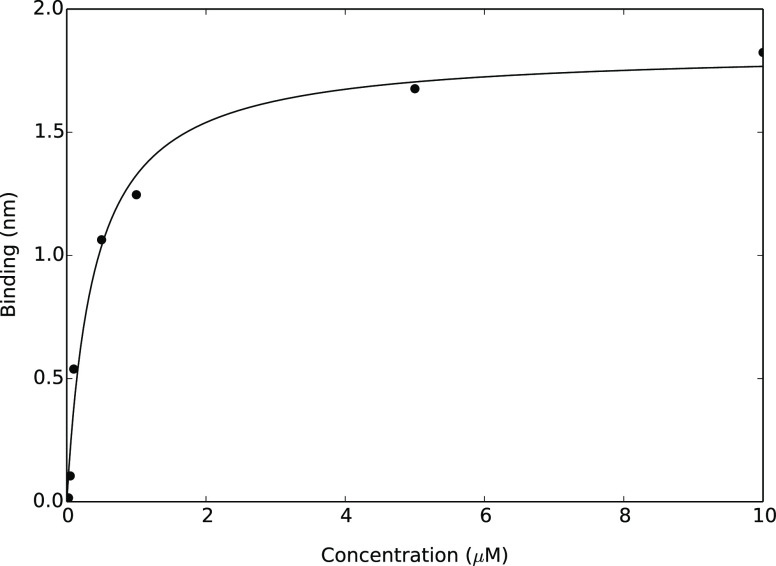
Langmuir isotherm for the adsorption of biotinylated aptamer 1C,5′ to a streptavidin-coated BLI tip. Each data point represents the maximum BLI shift observed once the system had reached equilibrium, and the fitted isotherm is characterized by the parameters *K*_A_ = 0.384 μM and *R*_max_ = 1.845 nm.

Biotinylated aptamer was adsorbed to streptavidin-coated BLI tips at a series of different concentrations to establish optimal conditions for achieving near-complete surface coverage. The resultant adsorption isotherm is illustrated in Figure 1. The experimental data points represent the maximum BLI shifts observed at each concentration, which are obtained once the system has reached equilibrium. Fitting to a Langmuir binding model (eq 1) reveals that a 5 μM aptamer solution is required to form a near-complete monolayer (>90% surface coverage) whereas ∼0.4 μM is required for 50% coverage.

**Figure 2:**
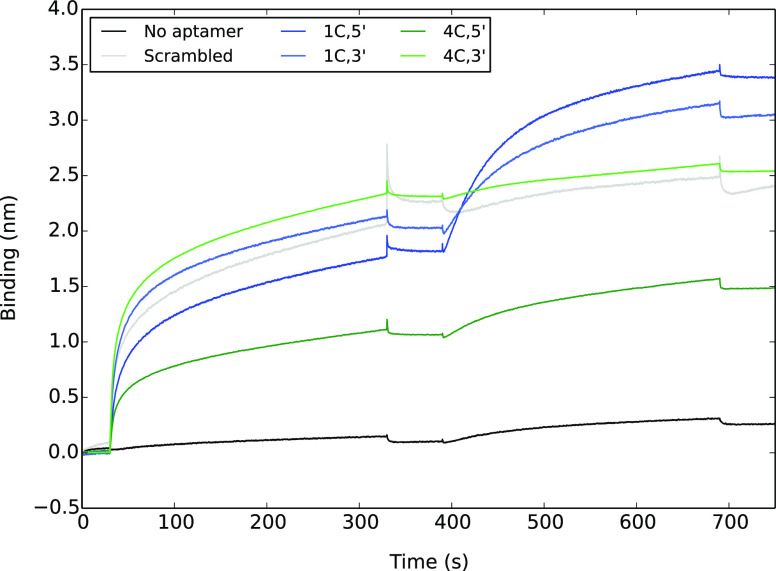
BLI binding curves for the adsorption of biotinylated aptamers to a streptavidin-coated surface at a concentration of 5 μM (30–330 s), which were then exposed to a 500 nM solution of spike protein (390–690 s). For details of the aptamer sequences, see the main text.

Surface immobilization of aptamers introduces orientational constraints that are not present in the solution-phase SELEX experiments from which the aptamer sequences were derived.^17^ We have pursued a two-pronged approach to circumvent this limitation: using two different sequences (1C, 4C) and attaching the biotin functional group to each end (3′, 5′) separately. This gives four different biotinylated aptamers that were each adsorbed onto BLI tips at a concentration of 5 μM. As a control, we also included an additional scrambled aptamer. Each loaded BLI tip was then immersed in 500 nM spike protein solution, producing the binding curves shown in Figure 2. Full details of our experimental procedure are available in the Materials and Methods section.

Although the aptamer concentrations are the same, the magnitudes of their BLI shifts are quite different. This is unlikely to be due to the different aptamers having different affinities for the surface because they all connect via the same biotin–streptavidin interaction. Instead, it is most likely due to different conformational and orientational preferences, leading to different biolayer thicknesses and changes in the surface refractive index. Surface packing effects may also alter surface concentrations.

Nonetheless, in all cases except the negative (no aptamer) control, the BLI responses are consistent with the aptamers covering half, or more, of the available sites, which provides a reasonable surface coverage to assess spike protein binding, particularly given that the trimer spike proteins are much larger than the immobilized aptamers.^18^

The 1C aptamers exhibit a significantly stronger BLI response upon spike protein binding than the 4C or scrambled control aptamers. In particular, the scrambled and 4C,3′ aptamers do not appear to bind the spike protein any more selectively or specifically than the unmodified surface, exhibiting binding curves very similar to the “no aptamer” negative control. Attaching the biotin on the 5′ end produces a larger BLI shift than the 3′ equivalent for both the 1C and 4C aptamers. On the basis of these results, the 1C,5′ aptamer (5 μM solution) was used as the only surface modifier in all subsequent work.

**Figure 3:**
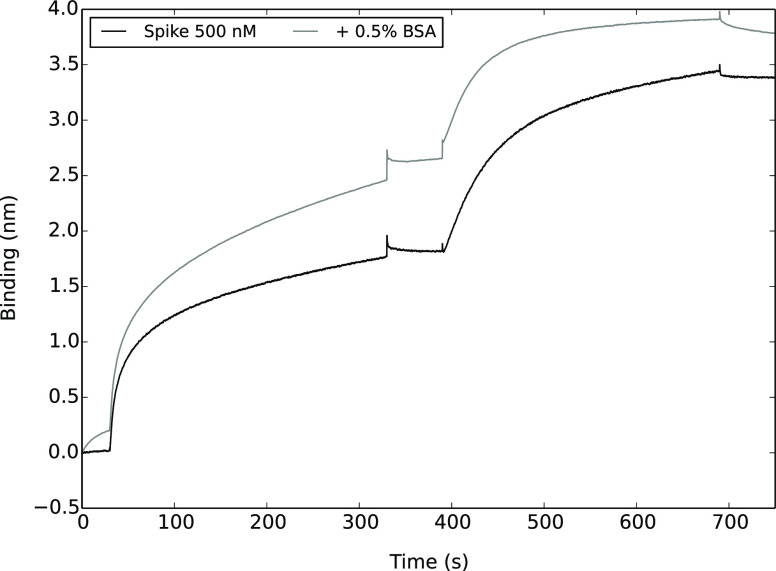
BLI binding curves for the adsorption of biotinylated aptamers to a streptavidin-coated surface at a concentration of 5 μM (30–330 s), followed by 500 nM spike protein solutions with and without BSA (390–690 s).

To test specificity of binding to the 1C,5′ aptamer, BLI binding curves were measured for 500 nM spike protein samples spiked with bovine serum albumin (BSA), as shown in Figure 3.

From these curves, it appears that the spike protein binding phase reaches equilibrium more rapidly in the presence of BSA, with a slightly lower equilibrium response. This is most likely due to BSA adsorbing non-specifically but not particularly selectively to the surface, blocking potential spike protein binding sites. This is consistent with previous studies, which have shown that measurement sensitivity decreases in biological media.^12,13^

**Figure 4:**
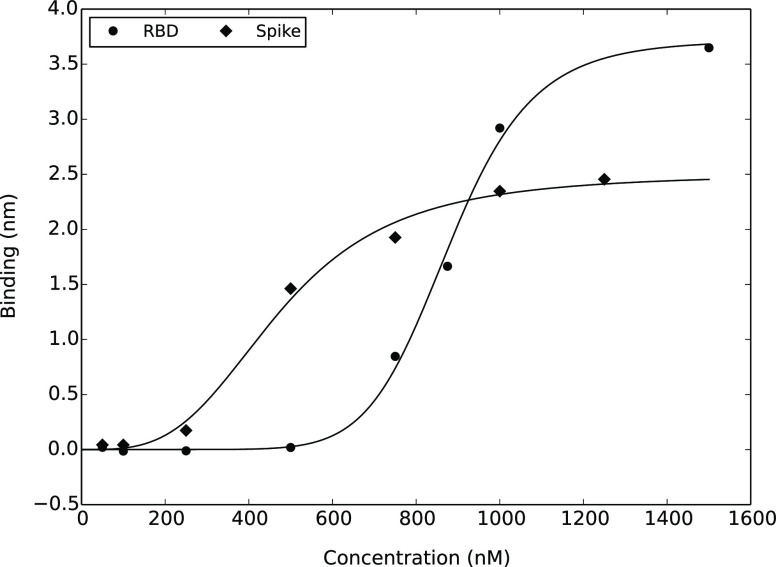
Equilibrium BLI shifts as a function of concentration for spike protein and its receptor-binding domain (RBD) binding to biotinylated 1C,5′ aptamer immobilized on streptavidin-coated BLI tips, fitted to the Hill equation (eq 2) using the parameters reported in Table 1.

To determine detection limits, binding curves were measured at a series of RBD and spike protein concentrations. Equilibrium BLI shifts are plotted as a function of concentration in Figure 4, and these data are fitted using the Hill equation (eq 2). At first glance, the results presented in Figure 4 and Table 1 are somewhat counterintuitive. The *smaller* RBD protein produces a *larger* maximum BLI shift, while the EC_50_ values for the two experiments are quite different, suggesting different binding affinities. However, the aptamers used in these experiments specifically target the receptor-binding domain,^17^ which is a subfragment of the full-length spike protein. Therefore, it is most likely that the spike protein binds to the aptamer through its RBD, with the same mode of binding and similar affinity. The only plausible explanation for these trends is therefore that the RBD protein forms aggregates at higher concentrations. This is also consistent with the fact that a higher Hill coefficient is required to characterize the fitted RBD model. Because of this tendency to form aggregates, the RBD protein is not a good model for “native” viral proteins. Therefore, only the spike protein is used in all subsequent work.

**Table 1:** Hill Equation Parameters that Characterize BLI Shifts Due to Binding of Spike Protein and Its Receptor Binding Domain to Surface-Immobilized Aptamers

parameter	spike	RBD
EC_50_ (nM)	474	879
*R*_max_ (nm)	2.50	3.72
*n*	3.37	8.77

Detection limits for spike protein binding to the immobilized 1C,5′ aptamer was computed from the blank (0 mM) binding curve as the mean absolute response value during the association phase plus associated one-sided confidence intervals^19^ at the 99th, 99.9th and 99.99th percentiles (Table 2). Unfortunately, the concentrations at which these detection limits are reached (∼250 nM) are not low enough to be practically useful in detecting viral particles at physiologically relevant levels. Respiratory fluid samples of COVID-19-infected individuals typically contain ∼7 × 10^6^ virions (viral particles) per mL,^20^ corresponding to a concentration of ∼0.01 pM or 10 fM. Even if the samples were preprocessed to break down the viral capsid and release the spike proteins into solution (24 ± 9 proteins per virion),^18^ this would still require a subpicomolar detection limit (∼0.25 pM).

**Table 2:** Limits of Detection for Spike Protein Binding to Biotinylated 1C,5′ Aptamer Immobilized on a BLI Tip, and the Concentrations at which This Limit Is Reached According to the Fitted Hill Model for Spike Protein Binding Response as a Function of Concentration

percentile	LOD (nm)	[spike] (nM)
99	0.09	236
99.9	0.10	250
99.99	0.12	260

The detection limit established here is broadly consistent with previous works that focus on developing BLI as a technique for in-process quantification of vaccine titer in which virus-like antigens are produced in relatively high concentrations. These studies report detection limits in the μg/mL range (∼50 nM).^21−23^

### Surface Plasmon Resonance (SPR)

SPR is a more sensitive technique for detecting and characterizing biomolecular interactions than BLI.^24^ It effectively measures changes in electrical permittivity through a thin surface layer of gold atoms upon analyte adsorption and, like BLI, allows kinetic (association and dissociation rate constants *k_a_* and *k_d_*) and thermodynamic (equilibrium dissociation constant *K*_D_) binding parameters to be concurrently determined.^25^ However, it does require substantially larger sample volumes than BLI, and experimental time frames are limited by maximum sample volumes and minimum flow rates.

**Figure 5:**
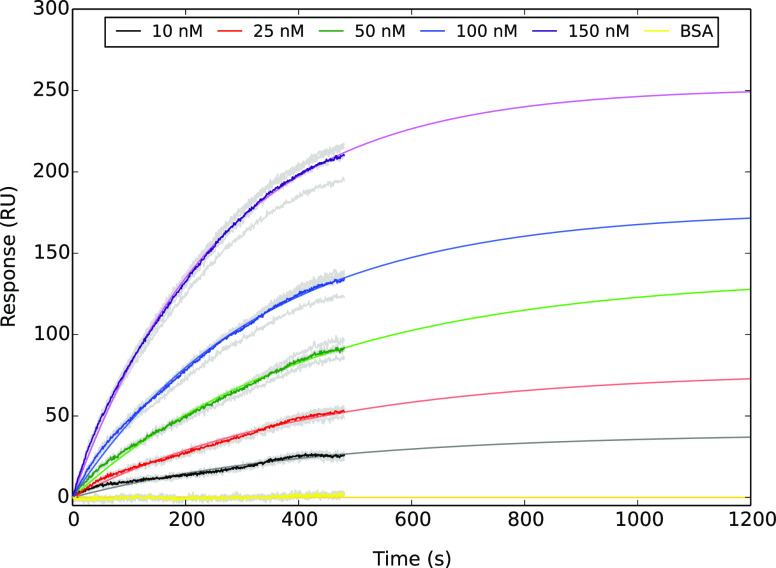
SPR sensorgrams for spike protein binding to thiolated 1C,5′ aptamer immobilized on a bare-gold SPR chip. Colored lines show averaged response curves based upon the raw data shown in gray, which are fitted to a first-order kinetic model. BSA control experiments were carried out at a concentration of 30 nM.

In this work, we are specifically interested in quantifying the detection limit for spike protein binding to thiolated aptamers immobilized on a gold surface. Therefore, it is not a particular problem that our sensorgrams do not reach thermodynamic equilibrium within the time frame of our experiments (Figure 5) because we are only looking to see whether there is a detectable difference from the BSA-only control.

**Figure 6:**
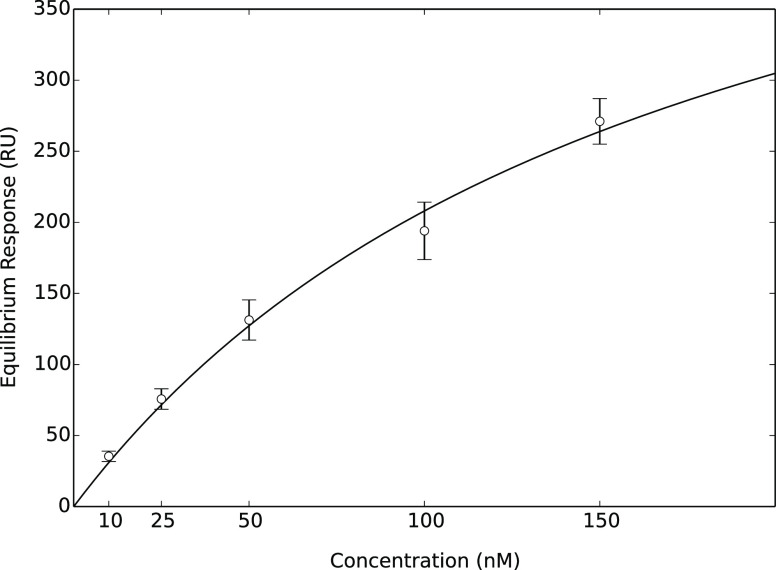
Average extrapolated equilibrium SPR responses at 10, 25, 50, 100, and 150 nM spike protein concentrations. Error bars represent the 95% confidence interval of the mean, obtained by fitting five replicates independently but keeping rate constants for lower concentration samples fixed at the values obtained at 100 nM. The fitted Hill model is characterized by the parameters *R*_eq, max_ = 570 nM, EC_50_ = 174 nM and *n* = 1.

From Figure 5, it is clear that the detection limit for aptamer-based sensing using SPR is comfortably below 10 nM. However, to obtain a more precise estimate, we must find a general relationship between analyte concentration and maximum SPR response. Extrapolated equilibrium response values are plotted as a function of concentration in Figure 6, and fitted using the Hill binding equation with *n* = 1.

Finally, it remains to quantify the detection limit for this technique. Analysis of the variability in the control (30 nM BSA) sensorgrams yields the one-sided confidence intervals reported in Table 3. According to fitted Hill model, concentrations in excess of 2.1 nM are required to produce extrapolated equilibrium responses larger than 6.7 RU, outside the variability within the BSA sensorgram. Therefore, we conservatively postulate that spike protein concentrations in excess of 5 nM will elicit SPR responses that are clearly distinguishable from the BSA-only control.

**Table 3:** Limits of Detection for Spike Protein Binding to Thiolated 1C,5′ Aptamer Immobilized on a Bare-Gold SPR Chip and the Concentrations at which these Limits Are Exceeded

percentile	LOD (RU)	[spike] (nM)
99	4.4	1.4
99.9	5.6	1.7
99.99	6.7	2.1

**Figure 7:**
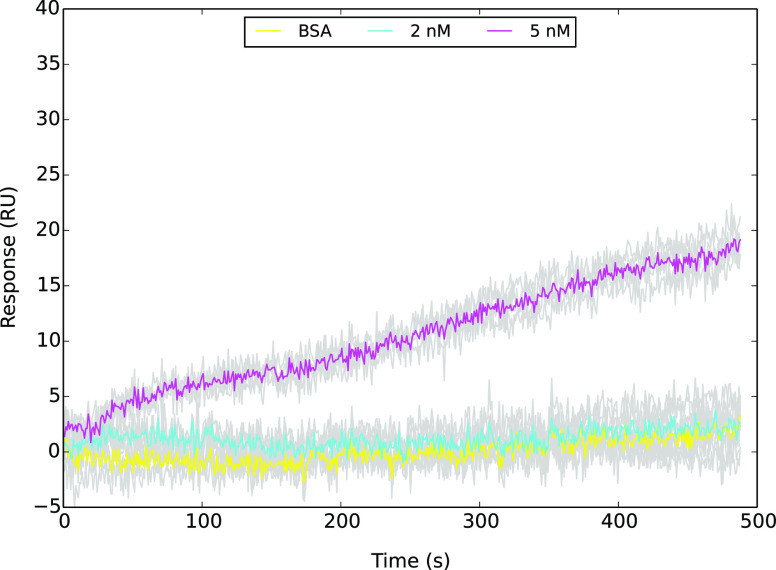
SPR sensorgrams for 2 and 5 nM spike protein binding to thiolated 1C,5′ aptamer immobilized on a bare-gold SPR chip. Colored lines show averaged response curves based upon the raw data shown in grey. BSA control experiments were carried out at a concentration of 30 nM.

This is borne out in the low concentration sensorgram data illustrated in Figure 7, in which the 5 nM samples can be clearly distinguished from the BSA baseline, whereas the 2 nM samples cannot. Although the signal to noise ratio at 5 nM is low and the binding curve quite flat, we are confident that this is a real response because it is consistently reproducible and also consistent with the response we would expect from the Hill isotherm model (predicted *R*_eq_ = 16 ± 4 RU).

Overall, our findings are consistent with previous studies, which have demonstrated low-nM detection limits for binding of viral proteins to oligonucleotide and/or antibody probes using commercial SPR machines.^26−29^ However, much lower detection limits have been reported for custom SPR systems.^15,30,31^

### Surface-Enhanced Raman Spectroscopy (SERS)

SERS is a very sensitive chemical detection method, allowing quantification of chemical and biological analytes to subpicomolar concentrations.^32,33^ It is based upon measuring the Raman spectrum of analytes that coordinate to the surface of nanoparticles or nanostructures, themselves immobilized on a solid support.^34^ In particular, the SLIPSERS method, which involves depositing nanoparticles onto a slippery, omniphobic substrate, is an ultrasensitive method that can detect analytes to subfemtomolar concentrations.^32^

However, its main limitation as a practical sensing technology is that the target analyte must coordinate selectively and specifically to the nanoparticles. Our strategy in this work is to use a thiolated aptamer that binds selectively and specifically to both the nanoparticle surface (through the thiol group) and also the target analyte (through the aptamer) and look for *changes* to the Raman spectrum of this probe biomolecule upon spike protein binding.

**Figure 8:**
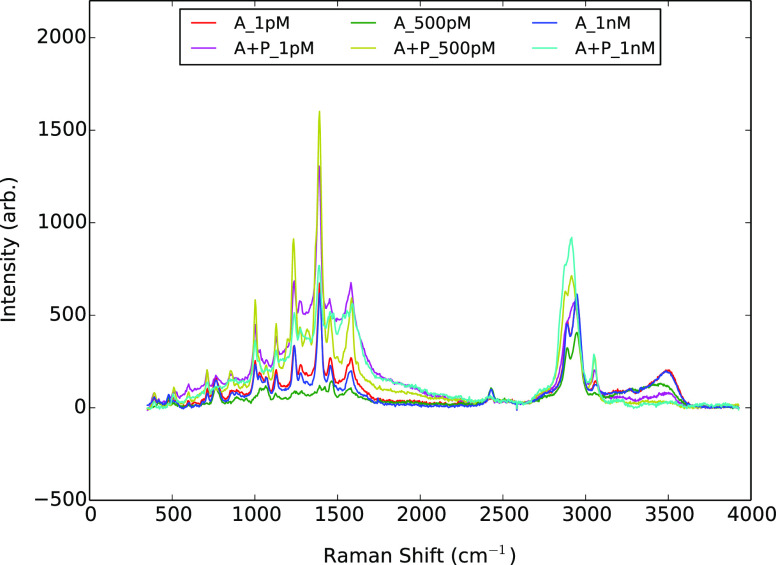
SERS spectra of aptamer (A) with and without spike protein (P) immobilized on silver nanoparticles and deposited on an omniphobic surface at a series of different concentrations. In all cases, a 1:1 stoichiometric ratio of aptamer to protein was used.

From the baseline-corrected spectra illustrated in Figure 8, it is evident that spike protein binding leads to depletion in the intensity of the broad N–H stretching band around 3500 cm^–1^ and a marked change in the band shape in the C–H stretching region (2800–3100 cm^–1^).

**Figure 9:**
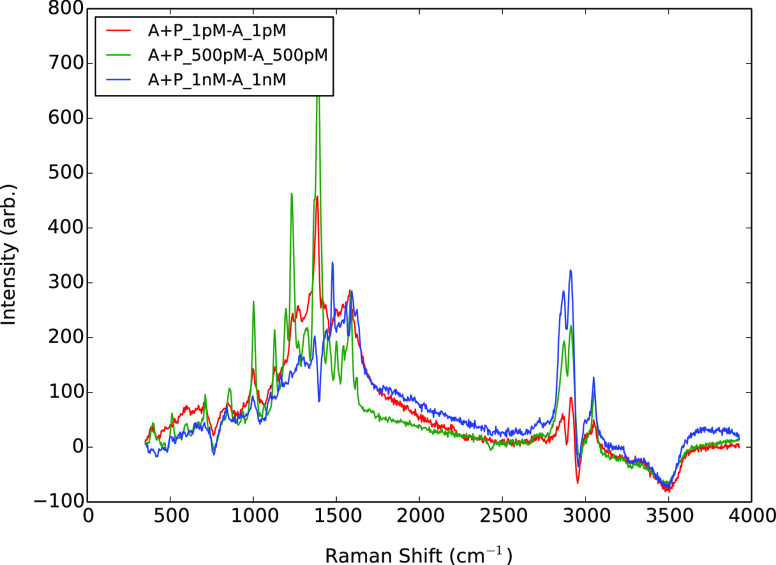
Principal component loadings that describe the majority of the variability between sets of SERS spectra for surface-immobilized aptamers with and without spike protein.

The spectral shift upon spike protein binding can be more clearly seen in the primary principal component loadings shown in Figure 9, which are obtained by analyzing variability across the combined data frames from the aptamer only and aptamer plus protein experiments at the same concentration. At all concentrations, not only does the broad N–H stretching band (3480 cm^–1^) from the aptamer spectrum decrease in absolute intensity, so too does the C–H stretching band at 2956 cm^–1^. The C–H stretching band at 2889 cm^–1^ also demonstrates a local dip in relative intensity. In place of these three depleted bands appear new red-shifted bands at 2870, 2912, and 3050 cm^–1^. This analysis also highlights the depletion of the small, sharp band at 763 cm^–1^ that is most likely a ring-puckering fundamental.^35^ Overall, this pattern of changes is consistent with peak shifting due to the formation of strong N–H···X bonds and much weaker C–H···X bonds between the immobilized aptamer and protein analyte.

**Figure 10:**
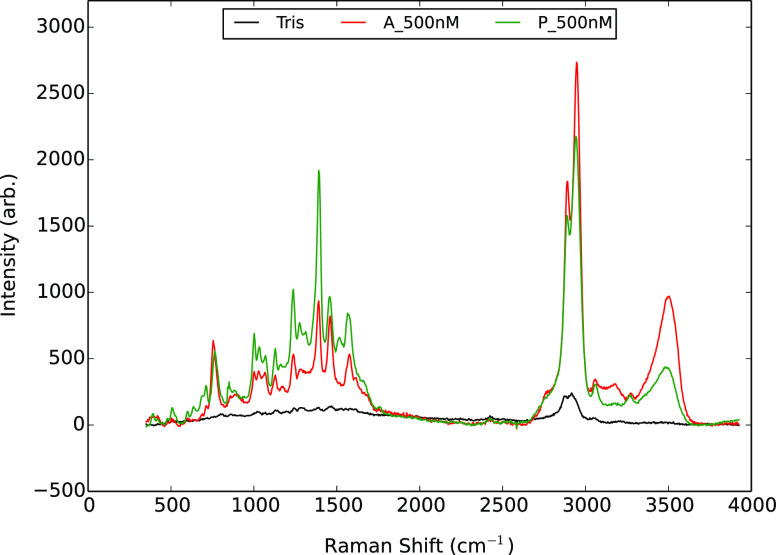
SERS spectra of silver nanoparticles treated with 500 nM aptamer (A) in Tris buffer, 500 nM spike protein (P) in Tris buffer, and Tris buffer alone (Tris) and deposited on an omniphobic surface.

Otherwise, the spectrum gains intensity in the so-called “fingerprint region” (750–1800 cm^–1^ for biomolecules^36^) but not in any particularly characteristic or concentration-dependent manner. This is most likely due to spike protein adsorbing non-specifically to exposed regions of the nanoparticle surface, in addition to binding through the aptamer and also superposed on the spectrum of the aptamer itself. Hence, the observed spectrum is a complicated mixture of all of these effects, which may be occurring in different ratios at different concentration regimes. Figure 10 shows that the spectrum of the spike protein directly adsorbed to the nanoparticle surface is very similar to that of the aptamer, supporting this interpretation and further confirming that the observed changes in the C–H and N–H stretching regions arise specifically from the formation of hydrogen bonds between the aptamer and spike protein only. Further, the depletion of the free N–H and C–H stretching bands is clearly a non-additive effect, again implying the formation of specific and selective interactions between the probe aptamer and spike protein analyte.

From Figures 8 and 9, we can conclude that there is a strong and reproducible shift in the SERS spectrum of the 1C,5′ aptamer upon spike protein binding down to a concentration of 1 pM, and likely substantially lower. For example, previous SERS studies have demonstrated subfemtomolar detection limits for recombinant viral proteins in phosphate-buffered saline,^37,38^ and one study even suggests that subattomolar detection of whole viral particles may be possible.^39^

**Figure 11:**
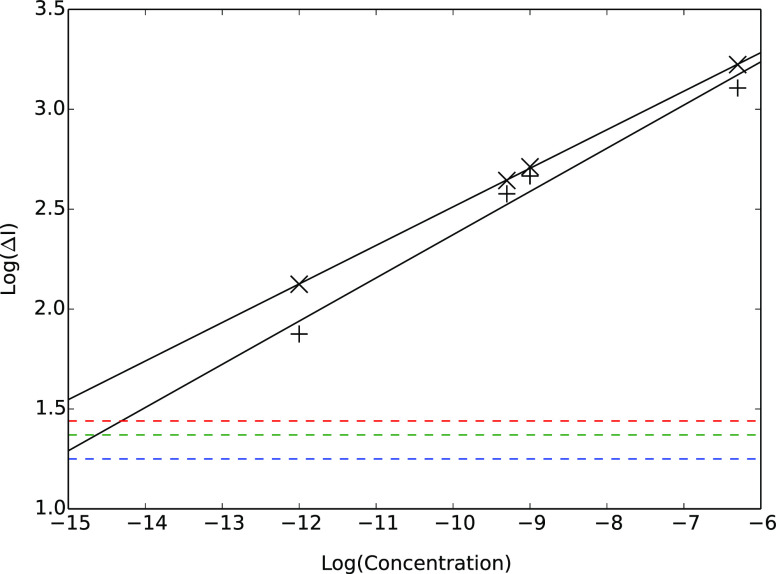
Differences in SERS intensities at 2872 cm^–1^ (crosses) and 2912 cm^–1^ (plus sign) between silver nanoparticles treated with aptamer alone (A) at a given concentration vs those treated with aptamer plus protein (A + P) at the same concentration. Detection limits are obtained by analysis of baseline scatter in the 3650–4000 cm^–1^ region. Dotted horizontal lines indicate 99, 99.9, and 99.99% one-sided confidence intervals.

From Figure 9, the most reliable concentration-dependent response is due to the appearance of new hydrogen-bonded C–H stretching modes at 2872 and 2912 cm^–1^. We have therefore reanalyzed our aptamer and aptamer-plus-protein spectra at 1 pM, 500 pM, 1 nM, and 500 nM and extracted changes in scattering intensity at these wavelengths, which are plotted as a function of concentration in Figure 11 on a log–log scale. Analysis of baseline variance in the 3650–4000 cm^–1^ region yields the detection limit intensities reported in Table 4 and indicated by dashed horizontal lines in Figure 11.

**Table 4:** Limits of Detection for Spike Protein Binding to Thiolated 1C,5′ Aptamer on Silver Nanoparticles by Monitoring Changes in SLIPSERS Spectra at 2872 cm^−1^, and the Concentrations at which these Limits Are Exceeded

percentile	Δ*I*_thresh_	log_10_(Δ*I*_thresh_)	log_10_([spike]) (M)	[spike] (fM)
99	18	1.25	–16.5	0.03
99.9	23	1.37	–15.9	0.13
99.99	28	1.44	–15.5	0.32

Monitoring changes in the Raman intensity at 2872 cm^–1^ appears to be the more reliable and sensitive approach, exhibiting a closer correlation between concentration and intensity change, slightly larger intensity changes, and a shallower line of best fit than observed at 2912 cm^–1^. This is presumably because this new band appears in a region of the spectrum that was otherwise featureless, whereas the band at 2912 cm^–1^ may overlap somewhat with the free C–H band at 2889 cm^–1^ that is concurrently depleted upon protein binding. From our calibration curve, changes in scattering intensity at 2872 cm^–1^ upon spike protein binding are expected to be differentiable from baseline scatter down to subfemtomolar concentrations, as reported in Table 4.

### Summary and Future Work

Of the three optical sensing techniques investigated in this work, only surface-enhanced Raman spectroscopy is sensitive enough to detect SARS-CoV-2 antigen proteins at physiologically relevant (sub-picomolar) levels. Analyte concentrations required to meet or exceed detection limits for all three approaches are summarized in Table 5. Detection limits for BLI and SPR are not limited by the affinity of the antigen for the oligonucleotide probe but rather reflect the inherent sensitivity of each technique.

**Table 5:** Concentrations of Spike Protein Required to Meet or Exceed the Detection Limit of each Analytical Method Investigated in this Work

technique	[spike]
BLI	250 nM
SPR	5 nM
SERS	1 fM

We have introduced a novel analysis technique to extract spectral shift profiles from SERS spectra and demonstrated that analyte binding can be visually identified by chemically meaningful depletion of characteristic peaks. In this case, depletion of the free N–H stretching band indicates formation of strong amide hydrogen bonds between the aptamer and its target protein. This is coupled to a more complex but characteristic set of spectral changes in the C–H stretching region that are also indicative of hydrogen bond formation. We have demonstrated a quantitative relationship between aptamer/analyte concentration and change in Raman scattering intensity at 2872 cm^−1^ due to the specific and selective formation of C–H hydrogen bonds between the aptamer and analyte.

This combination of SLIPSERS measurements using aptamer-functionalized silver nanoparticles and principal component analysis of the resultant spectra yields an overall detection methodology that is quick, sensitive, and applicable across a wide dynamic range (at least 6 orders of magnitude) and requires minimal sample volumes (10 μL). This makes it ideally suited as a foundation for developing new ultrarapid point-of-use diagnostics. One limitation is that a Raman spectrometer is required, although these can now be fabricated relatively cheaply (∼USD$1000).^40^

## Materials and Methods

### Materials

All aptamers used in this work (Table 6) were acquired from Integrated DNA Technologies.

**Table 6:** Aptamer Sequences Used in This Work

name	sequence and modifier	reference
1C,5′(biotin)	5′-biotin-CAGCACCGACCTTGTGCTTTGGGA	(17)
	GTGCTGGTCCAAGGGCGTTAATGGACA-3′	
1C,3′(biotin)	5’-CAGCACCGACCTTGTGCTTTGGGAGTGC	(17)
	TGGTCCAAGGGCGTTAATGGACA-biotin-3′	
4C,5′(biotin)	5′-biotin-ATCCAGAGTGACGCAGCATTTCATCGGGTCC	(17)
	AAAAGGGGCTGCTCGGGATTGCGGATATGGACACGT-3′	
4C,3′(biotin)	5’-ATCCAGAGTGACGCAGCATTTCATCGGGTCCAAAA	(17)
	GGGGCTGCTCGGGATTGCGGATATGGACACGT-biotin-3′	
scrambled (biotin)	5′-biotin-AACGCGGAGCCATTGGTAAGGTG	this work
	CGTCCGTCCTCAGTATCTAAGCTGTGGG-3′	
1C,5′(dithiol)	5′-dithiol-CAGCACCGACCTTGTGCTTTGGGA	(17)
	GTGCTGGTCCAAGGGCGTTAATGGACA-3′	
scrambled (dithiol)	5′-dithiol-AACGCGGAGCCATTGGTAAGGTG	this work
	CGTCCGTCCTCAGTATCTAAGCTGTGGG-3′	

Phosphate-buffered saline (PBS) was made up in milliQ water as a solution of 10 mM Na_2_PO_4_ (ECP Labchem), 1.8 mM KH_2_PO_4_ (ECP Labchem), 137 mM NaCl (Sigma Aldrich), and 2.7 mM KCl (Sigma Aldrich). To this solution, 0.005% by volume Tween-20 detergent (ThermoFisher Scientific) was added to form the SPR buffer (PBS-T). Tris buffer was made up as a 1 M solution of Tris base (ThermoFisher Scientific) in milliQ and adjusted to pH 8 using hydrochloric acid.

Analytical grade silver nitrate (99.8%, Aldrich), trisodium citrate (99.0%, BDH Chemicals), hydrogen peroxide (30% w/w, ThermoFisher Scientific), potassium bromide (99.0%, Ajax Finechem), and sodium borohydride (98.0%, Aldrich) were used in nanoparticle preparation.

### Recombinant Protein Production and Purification

A soluble version of the spike protein from SARS-CoV-2 isolate Wuhan-Hu-1 (GenBank: MN908947.3) was adapted from the design of Amanat *et al.*(41) The protein included a C-terminal T4 trimerization domain and hexahistidine tag, removal of the polybasic cleavage site (RRAR to A), and two stabilizing mutations (K986P and V987P) but lacked the thrombin cleavage site of the Amanat *et al.* construct. An RBD-only construct containing the native spike secretion signal (M1-Q14) and amino acids R319-F541, with a C-terminal polyhistidine tag was also designed. Sequence was codon-optimized for expression in human cells and synthesized by ThermoFisher and cloned into the pcDNA3.4 plasmid. The plasmid was purified using a NucleoBond Xtra Midi kit then transfected into Expi293F cells (ThermoFisher) using an ExpiFectamine 293 Transfection kit. Culture supernatants were harvested 3 days after transfection by centrifugation at 3000 × *g* and were clarified through a 0.45-μm syringe filter.

Culture supernatant was loaded onto a 5 mL HisTrap HP nickel sepharose column (Cytiva Life Sciences). Purified protein was eluted through a gradient of 0–400 mM imidazole in phosphate-buffered saline using an Akta Pure chromatography system (GE Life Sciences). Imidazole was removed by buffer exchange into PBS using a HiPrep 26/10 desalting column then the protein was concentrated using a Vivaspin 6 centrifugal concentrator with a molecular weight cutoff of 50,000. Protein purity was assessed by reducing SDS-PAGE and concentration was calculated using a NanoDrop ND-1000 UV–Vis spectrophotometer with predicted molecular weights of 137,512 and 25,921 Da and extinction coefficients of 142,835 and 33,850 for the spike protein and its receptor-binding domain, respectively. The protein was flash frozen in liquid nitrogen then stored at −80°C until use.

### Biolayer Interferometry

#### Setup

BLI experiments were performed on a Blitz instrument (Fortebio, USA) using the bundled Blitz Pro software. Prior to use, streptavidin (SA) functionalized biosensors (Fortebio, USA) were hydrated for 20 min in 200 μL phosphate-buffered saline (PBS), pH 7.4. Measurements were carried out at room temperature using the drop format with biotin-labeled aptamers, spike protein, and RBD diluted in PBS.

#### Aptamer Adsorption Isotherms

To determine optimal surface coverage of the SA-biosensors with the biotin-labeled aptamers, binding of the 1C,5′ aptamer was monitored as a function of time across a range of concentrations: 0, 0.025, 0.05, 0.1, 0.5, 1, 5, and 10 μM. Biosensor loading was performed as follows: (i) 4 μL PBS, baseline (30 s); (ii) 4 μL of diluted 1C,5′ aptamer, loading (300 s); (iii) 4 μL PBS, baseline (30 s).

#### Detection of SARS-CoV-2 Spike Protein and RBD Binding to Immobilized Aptamers

Spike protein and RBD binding to surface-immobilized aptamers (1C,5′, 1C,3′, 4C,5′, 4C,3′, scrambled) was monitored at a range of protein concentrations: 0, 0.05, 0.1, 0.5, 0.75, and 1 μM. Assay steps were the following: (i) 4 μL PBS, baseline (30 s); (ii) 4 μL of 5 μM biotin-aptamer, loading (300 s); (iii) 4 μL PBS, baseline (60 s); (iv) 4 μL diluted spike protein/RBD, association (600 s); and (v) 4 μL PBS, dissociation (60 s).

#### Data Analysis

At each concentration, equilibrium BLI shifts were measured as the difference of the values obtained at the start and end of the loading phase (aptamer immobilization) and association phase (protein binding), corrected for baseline shift using the corresponding value from the negative control (no aptamer/protein) experiment. For aptamer immobilization, these data were fitted to a Langmuir adsorption model:
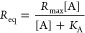
1where *R*_eq_ = equilibrium BLI response at a given concentration (shift in nm), *R*_max_ = maximum BLI response, [*A*] = aptamer concentration, and *K*_A_ = the association constant, which corresponds to the concentration at which 50% of the maximum response is obtained, indicating 50% surface coverage.

To describe protein binding as a function of concentration, the Hill equation is used:
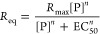
2where EC_50_ is the concentration at which half of the maximum response is observed and *n* is the dimensionless Hill coefficient.

Detection limit thresholds were computed as the mean absolute response of the blank (buffer only) sample plus one-sided confidence intervals at the 99th, 99.9th, and 99.99th percentiles.^19^

### Surface Plasmon Resonance

#### Chip Surface Functionalization

Chip surface functionalization was performed following the procedure of Wang *et al.*(42) A bare gold chip obtained from Bio-Rad was treated with piranha solution (concd H_2_SO_4_ + neat H_2_O_2_ in a 3:1 *v*/*v* ratio) and then rinsed thoroughly with milliQ water and ethanol. A solution of thiolated 1C,5′ aptamer (1 μmol L^–1^), 3-mercapto-1-propanol (10 μmol L^–1^), and dithiothreitol (0.01 mol L^–1^) was made up in Tris buffer (1 mol L^–1^, pH 8.0), spread on the surface of the chip, left overnight, and rinsed off thoroughly with milliQ water.

#### Interaction Measurements

SPR experiments were performed on a BioRad ProteOn XPR36 instrument (Bio-Rad, USA) using the associated ProteOn Manager software. Interaction binding curves were generated by monitoring the change in the SPR signal as a function of time for analyte flowing over the surface at a rate of 25 μL/min for a period of 480 s, followed by a 300 s regeneration step during which the chip was washed with a solution of 0.01 M glycine–HCl (pH 2.0) at a flow rate of 30 μL/min, followed by a washing step during which the chip was purged with PBS-T buffer for 300 s at 30 μL/min. Samples were run in batches of six in which each set contained one blank (SPR buffer only) and five samples at different spike protein concentrations, and responses were measured at six different points on the SPR chip. The five sensorgrams in closest concordance were analyzed together for each sample concentration. BSA control experiments were performed separately following the same procedure. Spike protein concentrations tested were: 1, 2, 5, 10, 25, 50, 100, and 150 nM.

#### Data Analysis

At high spike protein concentrations (>10 mM), baseline-corrected sensorgram curves were fitted to the kinetic model:
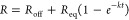
3

The relationship between protein concentration and association/dissociation rate coefficients was then determined by linear-least squares fitting to:
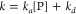
4

This equation was used to extrapolate rate coefficients at lower concentrations (<10 mM), where numerical fitting processes were unstable due to the relatively shallow nature of the sensorgram curves. Equilibrium responses at lower concentrations were then obtained by fitting to eq 3 using fixed extrapolated *k* values.

Detection limit thresholds were computed as the one-sided confidence interval of the mean response for the control (30 nM BSA) samples at the 99th, 99.9th, and 99.99th percentiles.^19^

### Surface-Enhanced Raman Spectroscopy

#### Preparation of the Nanoparticles

Silver nanoparticles were prepared following the method of Kitaev *et al.*(43,44) All solutions were made in MilliQ water. In a borosilicate 20 mL vial, 2.00 mL of trisodium citrate (1.0 × 10^–2^ M), 5.00 mL silver nitrate (3.75 × 10^–3^ M), 5.00 mL hydrogen peroxide (5.0 × 10^–2^ M), and 40 μL of potassium bromide (1 × 10^–3^ M) were added and stirred gently. Then, 2.5 mL of freshly prepared sodium borohydride (5.0 × 10^–3^ M) was added, and the vials were carefully swirled occasionally until no more color changes were observed (approximately 3 to 5 min), resulting in a stable yellow-colored solution.

#### Preparation of the SLIPS Substrates

SLIPS substrates were prepared following the method of Yang *et al.*(32) using a polyfluoropolyether oil, Krytox GPL105, and Sterlitech Polytetrafluoroethylene (PTFE) unlaminated Teflon membrane filters of 0.2 mm of pore size and a diameter of 13 mm. The Teflon membrane filters were placed on the center of a glass slide, and a drop of lubricant using a glass Pasteur pipette was dropped onto the center of the membrane filter. Glass slides were tilted so that the oil coated the entire membrane filter and then spun at 1500 rpm for approximately 1 min using a spin coater to ensure excess lubricant was removed.

#### Preparation of Spike Protein and Aptamer Samples

Thiolated aptamers, diluted to 100 μM in H_2_O, were reduced prior to sample preparation by adding Tris [2-carboxyethyl] phosphine (TCEP) solution (pH 7) to a final concentration of 10 mM and incubating the aptamers for 2 h at room temperature. The following concentrations of spike protein, thiolated aptamer (1C,5′), or equimolar mixtures of spike protein and thiolated aptamers were prepared in 50 mM Tris/HCl buffer (pH 8): 1 μM, 500 nM, 1 nM, 500 pM, and 1 pM. Spike protein binding to aptamers was facilitated by incubating mixtures for 15 min at room temperature.

#### Sample Loading

Ten microliters of the prepared samples or 50 mM Tris/HCl buffer (pH 8) only were added to 50 μL Ag colloidal solution and mixed thoroughly. The 60 μL mixtures were then pipetted onto the SLIPS surface, and the droplet was dried at 65 °C. Following evaporation of the solution, SERS measurements were performed on the aggregates, which were visible as small black dots.

#### Raman Spectroscopy

Raman spectra were recorded under ambient conditions with a custom-built Raman microscope.^45,46^ A 532 nm excitation (Laser Quantum Torus 532) was focused onto the silver nanoparticle/analyte aggregate via an N.A. = 0.65 (40× magnification) microscope objective. The laser power at the sample was <1 mW. Back-scattered Raman and Rayleigh scattered light was collected by the same objective, and the Rayleigh component was rejected by a 532 nm Raman edge filter (Iridian Spectral Technologies) and focused onto the entrance slit of a Teledyne (Princeton) Instruments Isoplane81 (FERGIE) spectrograph. Spectral data were acquired using LightField 6.1 software. No background removal was applied during data collection. The detector exposure time was 1 s and between 30 and 120 exposures (data frames) were captured and stored separately prior to data analysis.

#### Data Analysis

For each set of data frames, the variance-independent Raman spectrum was extracted as the primary principal component loading vector from an uncentered principal component analysis. Baseline correction was performed using the *derpsalsa* algorithm (λ = 1 × 10^6^), a modified form of asymmetric least-squares that uses derivative information to more appropriately interpolate smooth baselines across broad overlapping bands with extended tails.^47^

Variance between sets of data frames is also analyzed *via* principal component analysis, subtracting the baselined spectra from each set prior to analysis. To ensure that meaningful comparisons can be made between data sets with different numbers of exposures, covariance matrix elements for the smaller set are weighted according to the ratio between the number of frames in the larger set to the number of frames in the smaller set. The primary principal component loading vector gives the signature spectral shift; the coupled changes across the spectral profile that account for the majority of the variability between the two sets of data.
